# The mediating role of professional identity between role models and self-determination in medical students: A multicenter cross-sectional study

**DOI:** 10.1371/journal.pone.0337935

**Published:** 2025-12-19

**Authors:** Jingjing Liu, Jiayu Mou, Qing Wang, Fuying Li, Qinghua Zhao, Mingzhao Xiao, Yu You, Zhongchen Luo, Jiao Tang

**Affiliations:** 1 School of Nursing, Chongqing Medical University, Chongqing, China; 2 Department of Nursing, The First Affiliated Hospital of Chongqing Medical University, Chongqing, China; 3 Department of Urology, The First Affiliated Hospital of Chongqing Medical University, Chongqing, China; 4 School of Nursing, Guizhou Medical University, Guiyang, China; Mazandaran University of Medical Sciences, IRAN, ISLAMIC REPUBLIC OF

## Abstract

**Objective:**

This study aimed to investigate self-determination, explore the mediating role of professional identity between role models and self-determination, and detect how role models and professional identity contribute to the development of self-determination among medical students.

**Methods:**

A multicenter cross-sectional descriptive study involving 289 medical students in China was conducted. The Self-Determination Scale (SDS), Professional Identity Scale (PIS), and Sense of Admiration Scale (SAS) were employed to collect data. The bootstrapping method was used to analyze the mediating effect of professional identity between role models and self-determination.

**Results:**

The average score on the SDS was 71.21 (*SD* = 11.72), while the mean score on the PIS was 69.49 (*SD *= 11.57), and the mean score on the SAS was 61.48 (*SD *= 10.41). A high level of role model perception had a significant direct effect (40.54%) on the degree of self-determination among medical students. Furthermore, professional identity had a significant positive mediating effect (54.83%) on the relationship between role models and self-determination.

**Conclusion:**

Medical educators should focus on creating positive role models and enhancing their perceptions of these role models to foster self-determination among medical students. Additionally, strengthening professional identity could indirectly enhance the self-determination of medical students.

## Introduction

Self-determination is a recognized critical personality trait. It is defined as an individual’s capacity to make autonomous choices informed by a holistic understanding of personal needs and environmental contexts, along with the ability to act upon reflective decision-making processes [1–3]. The concept of self- determination originally emerged within the realm of special education for individuals with disabilities, aiming to enhance the quality of life and lifelong well-being of this population [4,5]. With the advent of positive psychology, an increasing number of scholars have focused on the significance of self-determination in fostering positive youth development, thereby promoting equitable outcomes in self-determination for students [5]. For instance, research has investigated the impact of self-determination on the motivation of college students and the learning capabilities of high school students [6,7]. The exploration of learning motivation and self-efficacy is a prevalent theme among medical students [8–10], while there is a conspicuous absence of research focused on the self-determination within this population.

In China, the decision to pursue a medical degree after the National College Entrance Examination is an intricate process, confronting dual pressures stemming from the unique educational and socio-cultural context. The National College Entrance Examination, often metaphorically described as “thousands of troops and horses crossing a single-log bridge”, represents a highly competitive and unavoidable gateway to higher education. Moreover, high-achieving students, in particular, face substantial familial and societal expectations regarding their career choices. Professions such as medicine—viewed as offering high prestige, stability, and financial security—are frequently promoted as ideal pathways, often leading students to prioritize external expectations over their intrinsic interests or passions.

Under the influence of the aforementioned pressures, the self-determination of high school students plays a significant role in their ability to autonomously select medical careers. Self-determination not only influences students’ choice of major but also significantly impacts their future career trajectories within the medical industry [11]. Medical students are poised to become the backbone of the future medical system, providing essential medical services to society [12]. Studies have revealed that during critical developmental stages such as adolescence and undergraduate education, enhanced self-determination significantly improves academic self-reliance, academic performance, social skills, and overall well-being [6,13,14]. High levels of self-determination during these developmental stages could even yield benefits in the workplace [15,16], such as reducing job burnout and eliminating stigmatization [17,18]. Given the importance of assessing self-determination levels among medical students, a reliable assessment tool was essential. To address this gap, we have adopted the widely recognized self-determination scale [19], which enables effective measurement and exploration of medical students’ self-determination levels, thereby supporting our hypothesis.

Previous studies have demonstrated strong correlations between professional identity, self-determination, and role models. Additionally, these studies indicated that a stronger professional identity might assist adolescents in making decisions about their future careers and enhance their understanding of workplace and career options [20,21]. Professional identity is operationalized as an individual’s cognitive, affective, and behavioral commitment to their future profession, as measured by the Professional Identity Scale (PIS) [22]. Through professional education, university instructors could encourage students to inspire career self-determination [22]. In recent years, the cultivation of a robust professional identity among medical providers has increasingly been recognized by medical educators as a central mission of medical education [23,24]. Medical professionals widely acknowledged that a heightened professional identity not only promotes favorable perceptions of the medical profession but also strengthens occupational prestige and accountability, while encouraging the development of positive professional conduct [24]. Scholars have extensively studied professional identity, revealing that medical professionals in China exhibited an average level of professional identity in China [25–27]. These studies indicated that medical students with higher professional identity were more likely to plan their career paths stably, pursue medical careers more resolutely, and experience greater satisfaction in their work. This sense of identity also fulfilled their need to contribute to society through their work, while simultaneously gaining recognition and respect [22,28–30]. Individuals with a robust professional identity were more inclined to make career decisions that result in positive outcomes [31–34]. Scales measuring professional identity are widely utilized in research. For this study, we selected a broadly applicable scale specifically designed for medical students to accurately assess their levels of professional identity [35].

Moreover, role models, as pedagogical strategies, have been extensively employed to enhance career motivation and professional excellence among medical students [36,37]. As behavioral archetypes, role models exert substantial influence on cognitive frameworks and decision-making patterns [38,39]. However, individual differences might result in either positive or negative responses to role models [40,41]. Recent research on role models has primarily concentrated on medical educators and students [37,40], underscoring their established significance within medical education curricula. Undergraduate students in the pharmacy and dentistry disciplines exhibited particularly strong susceptibility to the effects of role models, with teachers serving as pivotal figures in helping students internalize positive behaviors [42,43]. We believed that cultivating students’ self-determination through the observation of role models constituted a unique educational initiative [44,45]. Notably, the perceptions of role models among medical students have seldom been quantified. A role model admiration scale was recently developed in China to measure individuals’ admiration for role models via standardized scaling methods [46]. We have adopted this scale to gain deeper insights into the implications of role models for medical students’ professional skill development, learning motivation, interpersonal competencies, and future patient‒care relationships [46,47]. Research has confirmed that self-determination could be enhanced through the influence of external role models and the processes of observational learning [47,48]. In medical education, role models serve as a prerequisite for increasing the autonomy of medical students [39,49,50]. Additionally, while professional identity played a significant role in self- determination, evidence indicated that role models also contributed to shaping the professional identity of medical students [51].

In summary, our study revealed that the enhancement of self-determination among medical students was influenced by role models, with professional identity serving as a mediating factor in this relationship. Consequently, the hypotheses for this study were articulated as follows:

Hypothesis 1 – Professional identity and role models can influence the self-determination of medical students.
Hypothesis 2 – Professional identity has a mediating effect on the relationship between role models and self-determination in medical students.

## Methods

### Study design

This cross-sectional study was conducted from May 28 to June 28, 2022, recruiting medical students from universities recognized for their excellence in medical education across the southwestern provinces of Chongqing, Sichuan, Hunan, and Shanxi in China.

### Participants

The study focused on a cohort of dedicated full-time undergraduate students majoring in medical professions. The participants were enrolled via a nonprobability sampling technique known as convenience sampling. The inclusion criteria for this study required participants to be under 25 years of age, in accordance with the age restriction of the AIR Self-Determination Scale [19] and to be full-time undergraduate students enrolled in medical-related fields. The exclusion criterion was being on leave. Prior to the survey, the study was thoroughly explained to the participants, and written informed consent was obtained from the participants themselves, as all participants involved were adults (aged ≥18 years).

Awidely accepted guideline for determining the required sample size is to ensure a minimum of 10 participants for each variable [52]. In the present study, there were an estimated 20 variable, which necessitated at least 200 participants. A total of 383 questionnaires were distributed, yielding 289 valid responses (a reclamation rate of 75.4%), which were included in the statistical analysis.

### Data collection

Data were gathered via Questionnaire Star, a reputable digital survey instrument developed by Changsha Ranxing Information Technology. This platform enabled the distribution of 217 million surveys across China, resulting in a total of 17.41 billion responses. The methodology for data collection is detailed below.

#### E-questionnaire setting.

The survey was conducted on a digital platform, with a link provided for easy access via WeChat, China’s leading mobile messaging service. On the initial screen of the electronic questionnaire, participants were presented with an informed consent form that outlined their rights, including the freedom to participate or withdraw from the survey at any time without repercussions. After providing their consent, the participants navigated to the second page to assess their eligibility on the basis of predefined criteria. Researchers screened participants on the basis of their basic information to ensure that only those meeting the specified inclusion and exclusion criteria were invited to participate. Qualified individuals then proceeded to complete the electronic questionnaire, while others were directed to exit the link. The survey took participants approximately 5 minutes to complete.

#### E-questionnaire distribution.

Medical students were invited and encouraged to distribute the electronic questionnaire link to potential participants through their personal WeChat accounts and group chats. To enhance engagement, assistants responsible for disseminating the questionnaires were offered a financial compensation for their efforts. Additionally, participants were informed during the informed consent phase that they would receive compensation for completing the questionnaire; however, the specific nature of this compensation, such as random digital money via WeChat, was not disclosed in advance. This approach aimed to emphasize voluntary participation rather than the incentive itself. Consequently, the link was effectively disseminated across a broad network, facilitating widespread distribution. The questionnaire was easily accessible to participants through the shared link, allowing them to complete it by following the provided online instructions.

### Survey materials

Participants were required to complete a questionnaire consisting of a demographic data sheet (e.g., gender and grade), the Chinese version of the AIR Self-Determination Scale (SDS), the Professional Identity Scale (PIS), and the Sense of Admiration Scale (SAS).

#### Chinese version of the AIR Self-Determination Scale (SDS).

The Chinese version of the SDS was used in this study to evaluate self-determination among medical students. It has been previously employed to evaluate self-determination in both adolescents and adults [19]. The scale comprises 20 items distributed across four dimensions: Things I do (TID), How I feel (HIF), What Happens at School (WHS) and What Happens at Home (WHH). The “Things I Do” dimension primarily assesses students’ understanding of their own circumstances; “How I feel” focuses on the inner emotions of the students; “What Happens at School” evaluates students’ self-determination capabilities within an academic setting; and “What Happens at Home” examines students’ self-determination at home, including the support received from family members. Responses to these items are measured via a 5-point Likert scale. Both the original AIR and its Chinese version have demonstrated adequate reliability and validity, with the Chinese version of the SDS reporting a Cronbach’s alpha coefficient of 0.937 [19]. In this sample, the Cronbach’s alpha coefficient for the SDS was found to be 0.940.

#### Professional Identity Scale (PIS).

The PIS, developed by Hao [35] in 2011, was utilized to assess the professional identity of medical students. This scale comprises three dimensions with a total of 19 items: professional self-awareness (PSA), autonomy of career choices and career persistence (AC), and occupational exploration behavior (OEB). Each item is rated on a 5-point Likert scale, with total scores for the PIS ranging from 19–95. The Cronbach’s alpha for the Chinese version of the PIS was reported to be 0.926 [35,51]. In our study, the Cronbach’s alpha coefficient for the PIS was found to be 0.827.

#### Sense of Admiration Scale (SAS).

The SAS, originally developed by Chen [46], was used to measure role models. This instrument has been utilized in China to gauge students’ perceptions of role models [48,49], demonstrating a high level of internal consistency, with a Cronbach’s alpha of 0.942. It consists of 15 items categorized into three dimensions: ability admiration (AA), virtue admiration (VA), and sense of worship (SOW). Each item is rated on a 5-point Likert scale, with total scores ranging from 15 to 75. A higher total score reflects a greater perception of role models. In our study, the Cronbach’s alpha coefficient for the SAS was 0.966.

### Statistical analysis

IBM SPSS Statistics for Windows, version 25.0 (IBM Corp., Armonk, N.Y., USA), was used to analyze the collected data. Descriptive statistics were used to express the sample characteristics, SDS, PIS, and SAS. Independent-sample t tests and one-way analysis of variance (ANOVA) were conducted to compare differences in SDS, PIS, and SAS (continuous variables in this study showing a normal distribution). Pearson correlation analysis was performed to test the relationships among SDS, PIS, and SAS, alongside significant main effects. The statistical significance of the mediating effect was subsequently verified via a bootstrapping method with the SPSS PROCESS macro (Model 4, version 4.0, developed by Andrew F. Hayes) [53,54]. Currently, the preferred approach for testing indirect effects is through a bootstrapped confidence interval. Bootstrapping, as applied in mediation analysis, involves resampling the data a specified number of times (e.g., 5000), allowing for the assessment of the indirect effect within each sample. Specifically, to determine whether professional identity explains the association between self-determination and role models among medical students, we calculated a 95% confidence interval (CI) via 5000 bootstrapped resamples. If zero does not fall within the 95% CI, there is a 95% likelihood that the indirect effect is significant. Statistical significance was established at P < 0.05.

### Ethical approval

This study involving human participants received approval from the Medical Ethics Committee of Chongqing Medical University (No: 2022106) in accordance with the Guideline for the Administration and Ethical Review of Ethics Committees in Biomedical Research of China and the ethical principles outlined in the Declaration of Helsinki, as revised in 2024. All participants were informed about the study’s background, purposes, procedures, potential benefits and risks, as well as privacy protection protocols, and informed consent was obtained.

## Results

### Demographic characteristics

The demographic characteristics of the participants are summarized in Table 1. The participants had a mean age of 19.85 years (*SD = *1.40). The majority of participants were female (*n = *208, 71.9%) and in Grade 2 or below (*n = *210, 72.6%).

**Table 1:** Demographic characteristics and group differences in SDS, SAS, and PIS.

		SDS			SAS			PIS		
Characteristic	*n (%)*	Mean±SD	*t* or *F*	*P*	Mean±SD	*t* or *F*	*P*	Mean±SD	*t* or *F*	*P*
**Gender**			1.327	0.186^a^		−0.716	0.475^a^		3.450	0.001^^a^*^
Female	208(71.9)	72.51 ± 1.36			60.60 ± 1.28			68.05 ± 11.45		
Male	81(28.1)	70.59 ± 0.79			61.76 ± 0.69			73.18 ± 11.10		
**Grade**			2.628	0.051^b^		3.405	0.018^^b^*^		3.859	0.009^^b^*^
Freshman	113(39.1)	73.51 ± 11.71			62.92 ± 10.76			72.00 ± 11.42		
Sophomore	97(33.5)	70.27 ± 12.08			62.05 ± 9.59			68.76 ± 11.95		
Junior	51(17.6)	69.47 ± 10.06			59.94 ± 10.24			67.88 ± 10.44		
Senior	28(9.8)	68.35 ± 12.25			61.48 ± 10.41			64.82 ± 10.99		
**First-level discipline**			1.319	0.269^b^		0.606	0.546^b^		8.207	0.0003^^b^*^
Clinical medicine	129(44.6)	71.93 ± 12.40			61.16 ± 10.88			72.48 ± 11.23		
Nursing	103(35.6)	71.55 ± 10.77			62.34 ± 9.73			67.06 ± 11.09		
Others^c^	57(19.8)	68.98 ± 11.71			60.63 ± 10.60			67.08 ± 11.76		

Note: SDS, Chinese version of the AIR self-determination scale; SAS, Sense of Admiration Scale; PIS, Professional identity scale; SD, standard deviation.

^a^Statistic was based on independent samples t-tests.

^b^Statistic was based on one-way analysis of variance (ANOVA).

^c^Others include public health & preventive medicine, medicine technology, traditional Chinese medicine, preclinical medicine, stomatology, pharmacy, integrated traditional Chinese and Western medicine, and traditional Chinese pharmacology.

*P < 0.05.

### SDS, PIS, and SAS scores of medical students

The average score on the SDS was 71.21 (*SD *= 11.72). Among the dimensions assessed, “What Happens at School” recorded the lowest score of 13.81 (*SD *= 2.81), while “What Happens at Home” exhibited the highest score of 21.15 (*SD* = 4.29). In terms of professional identity, the mean score for PIS was 69.49 (*SD *= 11.57), which included scores of 26.12 (*SD *= 4.83) for the “Occupational Exploration Behavior” dimension, 29.45 (*SD *= 6.17) for the “Professional Self-Awareness” dimension, and 13.91 (*SD *= 2.34) for the “Autonomy of Career Choices and Career Persistence” dimension. Additionally, the mean score for SAS was 61.48 (*SD *= 10.41), comprising scores of 24.77 (*SD *= 4.30) for the “Ability Admiration” dimension, 16.63 (*SD *= 2.89) for the “Virtue Admiration” dimension, and 20.06 (*SD *= 3.79) for the “Sense of Worship” dimension (Table 2).

**Table 2:** Descriptive summary and correlation analysis of PIS, SAS, and SDS.

Variables	Mean±SD	SDS	
		*r*	*P*
**SDS**	71.21 ± 11.72		
Things I do	21.40 ± 3.78		
How I feel	14.84 ± 2.45		
What happens at school	13.81 ± 2.81		
What happens at home	21.15 ± 4.29		
**PIS**	69.49 ± 11.57	0.714^**^	＜0.01
Professional self-awareness	29.45 ± 6.168	0.590^**^	＜0.01
Autonomy of career choices and career persistence	13.91 ± 2.34	0.454^**^	＜0.01
Occupational exploration behavior	26.12 ± 4.83	0.735^**^	＜0.01
**SAS**	61.48 ± 10.41	0.600^**^	＜0.01
Ability admiration	24.77 ± 4.30	0.569^**^	＜0.01
Virtue admiration	16.63 ± 2.89	0.506^**^	＜0.01
Sense of worship	20.06 ± 3.79	0.605^**^	＜0.01

Note: SDS, Chinese version of the AIR self-determination scale; PIS, Professional identity scale; SAS, Sense of Admiration Scale; ^**^P < 0.01.

### Differences in SDS, SAS, and PIS among various demographic characteristics sub-groups

The univariate analysis revealed that gender (*P* ＞ 0.05) and first-level discipline (*P* ＞ 0.05) were not significantly associated with self-determination and role models (Table 1). However, gender (*t *= 3.450, *P *< 0.01), grade (*F *= 3.859, *P *< 0.01), and first-level discipline (*F *= 8.207, *P *< 0.01) showed significant associations with professional identity.

### Correlations between SDS, PIS and SAS

The SDS had a positive and strong relationship with PIS (*r = *0.714, *P < *0.01) and SAS (*r = *0.600, *P < *0.01). (Table 2). Additionally, the SDS was positively correlated with the PIS and SAS subscales, such as “Occupational Exploration Behavior” (*r *= 0.735, *P *< 0.01), “Ability Admiration” (*r *= 0.569, *P *< 0.01), and “Virtue Admiration” (*r *= 0.506, *P *< 0.01) (Table 2).

### Mediating effect of professional identity

**Fig 1:**
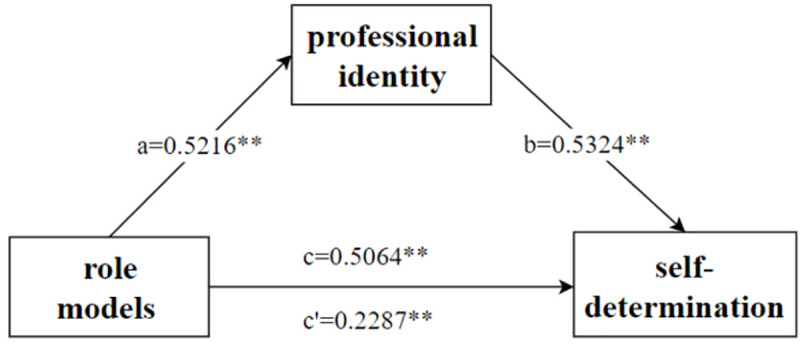
The mediating role of professional identity between role models and self-determination.

Tables 3 and 4 present the results of the mediating analysis, where all covariates were controlled for, and bootstrapping methods were employed. First, the findings indicated that role models positively influenced professional identity (*β = *0.590, *P <* 0.001). Second, role model had a positive predictive effect on self-determination (*β = *0.591, *P <* 0.001). Finally, professional identity served as a partial mediator between role model and self-determination (Fig 1), with an indirect effect of 0.2777, which was statistically significant and accounted for 54.83% of the total effect *(95% CI:* 0.2078–0.3589*, P < *0.001).

**Table 3:** Mediating effects of PIS between SAS and SDS.

Step	Pathway	B	SE	β	t	Adj.R^2^	F
1	Role models →Professional identity	0.518	0.041	0.590	12.740^***^	0.403	49.684
2	Role models →Self-determination	0.499	0.040	0.591	12.375^***^	0.368	42.868
3	Role models →Self-determination	0.218	0.042	0.259	5.155^***^	0.555	72.943
	Professional identity →Self-determination	0.542	0.049	0.563	10.994^***^		

Note: B, unstandardized regression coefficient; SE, standard error; β, standardized regression coefficient; Adj.R^2^, Adjusted coefficient of determination; ^***^P < 0.001.

**Table 4:** Verifying the bootstrapping mediation effect.

Professional identity	B	SE	Bootstrap 95% CI	Proportion of effect
Indirect effect	0.2777	0.0387	0.2078, 0.3589	54.83%
Direct effect	0.2287	0.0413	0.1420, 0.3205	40.54%
Total effect	0.5064	0.0400	0.4280, 0.5850	

Note: B, unstandardized regression coefficient; SE, standard error; CI, confidence interval.

## Discussion

The gender distribution of medical students in this study appeared imbalanced; however, it accurately reflected the current reality of higher education in China. According to the gender ratio of undergraduate students published by the Ministry of Education of China from 2013 to 2021, the proportion of female students has consistently exceeded that of male students, demonstrating a continuous upward trend [55]. Furthermore, essentialist views on gender suggested that males and females possess different interests and strengths across various fields of study; for instance, males were generally more adept at logical reasoning, while females excel in memorization [56]. Additionally, societal perceptions of gender roles imposed distinct demands for the social division of labor on both genders, resulting in males and females tending to select different majors. This contributed to a prevalent phenomenon of gender segregation in medical education in China, characterized by a high concentration of professionals of a particular gender within specific specialties [56,57]. For example, a study in China reported that the proportion of female students in nursing reached as high as 84.5% [58]. In this context, this study explored the effects of role models on self-determination among medical students. A path model was constructed to elucidate the direct and indirect effects between variable pairs, mediated by professional identity. The results showed that role models significantly influenced self-determination, with professional identity serving as a partial mediator in the relationship between role models and self-determination among medical students.

This study revealed an overall moderate level of self-determination among medical students, consistent with self-determination levels reported in previous studies on college students [16,17,19], but slightly higher than those observed in disabled adolescents [4,5]. Furthermore, medical students achieved the highest scores in the “What Happens at Home” dimension, suggesting a strong level of self-determination at home and robust support from family members. These findings might be attributed to the substantial family and social support that medical students received in China [59], as well as their heightened sense of self-efficacy and well-developed self-management skills [60].

Nevertheless, medical students performed the worst in the aspect of “What Happens at School”. This domain primarily measured students’ self-determination abilities within the educational setting. This phenomenon differed from the factors contributing to the lower self-determination levels observed among other college [19] and disabled students [4,5,61]. First, Upon entering university, medical students often encounter a more rigorous learning environment and heightened academic demands, making the transition particularly challenging as they confront complex content and elevated expectations [62,63]. Second, medical students, having recently transitioned from the high-pressure environment of high school, find themselves immersed in a demanding curriculum. They must navigate a wide array of subjects, ranging from basic medical sciences to clinical practice, while managing frequent exams and assessments [62]. Third, medical education typically adheres to a rigid curriculum with limited flexibility. Students often have minimal choices regarding course selection or the pacing of their learning, which can diminish their sense of autonomy and self-determination [64]. Finally, in China, the medical profession is perceived as both high-stakes and high-rewards. The significant expectations placed on these students can create substantial pressure, compelling them to excel academically. This pressure may lead to tension and discomfort within the school environment, ultimately impacting their performance and self-determination [65]. Hence, offering incoming medical students theoretical courses on emotional and stress management to equip them with the necessary skills to navigate their academic journeys is essential.

In this study, role models were identified as a significant factor influencing medical students’ self-determination. For the medical students in this sample, achieving a high level of self-determination through “Virtue Admiration” was deemed essential. This phenomenon is closely linked to China’s rich tradition of medical ethics education [46,47]and the moral expectations placed upon medical professionals. In China, society enforces nearly obligatory moral standards on medical workers, and this prevailing value system profoundly shapes medical students’ identification with these moral exemplars. Notably, role models for medical students should extend beyond on-campus learning to include clinical practice [42,43,47,66]. Establishing medical role models who are both virtuous and skilled within the complex clinical environment could profoundly influence medical students’ career planning and life choices [31,32], thereby reflecting an enhancement in their levels of self- determination.

This study revealed a positive correlation between medical students’ professional identity and self-determination. medical students with greater professional identity tended to exhibit greater self-determination, likely due to several interrelated factors. First, their strong intrinsic motivation, stemming from deep profound medical and a sense of mission, drived them to engage more proactively in both learning and practice [65]. Second, these students possessed clear career goals, which provided them with direction and purpose, thereby enhancing their self- determination [31,32,67]. Additionally, their heightened self-efficacy, rooted in confidence in their abilities, further strengthened their decision-making autonomy [68]. Finally, they focused on long-term career development allowed them to connect current efforts with future aspirations, making them more proactive and strategic in their career planning [32,33,69]. Medical students with a heightened sense of professional identity exhibit increased levels of self-determination, which in turn fueled their engagement in long-term career planning. This proactive approach not only benefited their personal growth but also positively impacted the advancement of the medical sector [70,71].

The professional identity of medical students aligned with prior studies [25–27], revealing a distinction between the professional identities of clinical medicine students and nursing students [27]. Medical schools should offer targeted career planning courses to assist students in defining their career goals and developing actionable pathways, while emphasizing the importance of long-term career development to align academic learning with future professional aspirations. Mediation analysis has confirmed the mediating role of professional identity in the relationship between role models and self-determination. When professional identity was incorporated into the regression equation, the predictive power of role models on the self-determination of medical students was reduced, indicating that professional identity serves as a mediator [72]. Enhancing professional identity, leveraging the inspiration from role models, and fostering self-determination in medical education can significantly advance students’ professional development and autonomy.

## Limitations

The present study had several limitations. First, the cross-sectional design restricted the ability to establish causal relationships among role models, professional identity, and self-determination. Future research could adopt a cohort study design to explore the causal relationships among these three variables more effectively. Second, the use of convenience sampling from a specific region (Southwestern China) limited the generalizability of the findings, despite the multi-center design. Future studies should consider employing stratified sampling to enhance the representativeness of the sample. Third, the cultural context of the study need to be considered, as the findings might not be fully applicable to other countries. Factors such as strong family support, a significant emphasis on medical ethics, and the high-pressure pre-university education environment in China may have influenced the outcomes. Future cross-cultural studies are necessary to examine the generalizability of these finding.

## Conclusions

This study revealed a moderate level of self-determination among medical students. Role models and professional identity positively influenced medical students’ self-determination, with professional identity serving as a mediating variable between role models and self-determination. This perspective introduced a novel approach to medical education, highlighting the importance of enhancing medical students’ self-determination through the improvement of role models and the development of professional identity, which subsequently facilitated their career advancement. In the context of medical education in China, prioritizing the enhancement of professional identity among medical students was imperative. To address this goal, it was essential to introduce more courses focused on the development of professional identity, enabling medical students to gain a comprehensive understanding of the roles and responsibilities of medical professionals from diverse perspectives. Medical colleges should offer courses on emotional and stress management, highlight virtuous and competent role models, and provide targeted career planning to strengthen professional identity. Furthermore, curriculum reforms should emphasize flexibility to foster autonomy, while leveraging family and social support can enhance self-efficacy. These measures aim to empower students, thereby supporting their academic and professional growth.
